# Effects of Huang Qi Decoction on Endothelial Dysfunction Induced by Homocysteine

**DOI:** 10.1155/2016/7272694

**Published:** 2016-09-20

**Authors:** Shuang Chu, Xiao-dong Mao, Li Wang, Wen Peng

**Affiliations:** ^1^Laboratory of Renal Disease, Putuo Hospital, Shanghai University of Traditional Chinese Medicine, Shanghai, China; ^2^Department of Nephrology, Putuo Hospital, Shanghai University of Traditional Chinese Medicine, Shanghai, China

## Abstract

Vascular endothelial dysfunction can be induced by homocysteine (Hcy) through promoted oxidative stress. Huang Qi decoction (HQD) is a traditional Chinese medical formula and its components possess antioxidant effect. The study herein was therefore designed to investigate the effects of HQD at different dosage on endothelial dysfunction induced by Hcy. Tempol and apocynin were used to investigate whether antioxidant mechanisms were involved. Endothelium-dependent relaxation of rat aortas was investigated by isometric tension recordings. Reactive oxygen species (ROS) in human umbilical vein endothelial cells (HUVECs) was determined by DHE staining. The assessment related to oxidative stress and NO bioavailability was performed by assay kits and western blot. In isometric tension experiment, HQD at the dose of 30 or 100 *μ*g/mL, tempol, or apocynin prevented impaired endothelium-dependent relaxation in isolated aortas elicited by Hcy. In cellular experiments, substantial enhancement in NADPH oxidase and ROS generation and reduction in NO bioavailability triggered by Hcy were reversed by pretreatment of HQD at the dose of 100 *μ*g/mL, tempol, or apocynin. The results proved that HQD at an appropriate dosage presented favorable effects on endothelial dysfunction initiated by Hcy through antioxidant mechanisms. HQD can act as a potent prescription for the treatment of endothelium related vascular complications.

## 1. Introduction

Endothelial dysfunction, featured by an impaired endothelium-dependent vasodilatation, has been considered to be involved in the pathogenesis of diabetes mellitus and related vascular complications [1]. Oxidative stress is one of the risk factors that have been found to contribute to vascular endothelial dysfunction and thereby the development of diabetic vascular syndrome [2, 3]. Inordinately increased plasma homocysteine (Hcy), a situation named hyperhomocysteinemia (Hhcy), is documented to be associated with vascular endothelial dysfunction by impairing nitric oxide- (NO-) mediated endothelium-dependent relaxation of large arteries [4]. Hcy has been suspected to promote oxidative stress in endothelial cells through overgeneration of reactive oxygen species (ROS), which partially stems from nicotinamide adenine dinucleotide phosphate (NADPH) oxidase. Then excessively generated ROS would severely restrict the bioavailability of endothelium derived NO, the important regulator of endothelial function in diabetic vascular complications which caused relaxation of vascular muscle layer [5]. Accordingly, decreasing ROS in endothelial cells offers an appealing strategy to normalize Hcy-induced impaired endothelium-dependent vasomotor response and endothelial dysfunction.

Huang Qi decoction (HQD) is a classic traditional Chinese medical formulation consisting of various medicinal herbs, which is first described thousands of years ago. It is comprised of* Astragalus* (Huang Qi), Poria (Fu Ling),* Trichosanthes* (Gua Lou),* Ophiopogon* (Mai Dong),* Schisandra* (Wu Wei Zi), licorice (Gan Cao), and* Rehmannia* (Di Huang). Accumulative evidence has revealed that components of HQD possess antioxidant activities or could improve diabetes related syndrome.* Astragalus* possesses rich antidiabetic active ingredients such as Astragaloside IV and holds potential for alleviating glucose intolerance and hypertriglyceridemia in DM and improving metabolic syndrome and endothelium dysfunction in fructose-fed rats [6]. Wang et al. demonstrate the antioxidant capacity of carboxymethyl (1→3)-*β*-D-glucan sulfate from Poria [7]. Liuwei Di Huang, a traditional Chinese herbal formula containing* Rehmannia*, is demonstrated to suppress chronic inflammation and oxidative stress in obese rats [8]. Triterpenoid or 5-hydroxymethylfurfural from* Schisandra* is reported to have favorable antioxidant effects on alcoholic liver oxidative injury [9, 10]. A report demonstrates that Gua Lou Guizhi decoction exerts neuroprotective effect by enhancing endogenous antioxidant enzymatic activities [11]. Accordingly, HQD has a potential antioxidant capacity to improve endothelial dysfunction. Actually, the active compounds in the constituent herbs of HQD have been reported to exert an influence on the vascular endothelial dysfunction, abnormal vasodilation, or vascular reactivity. Catalpol found in* Rehmannia* and Ophiopogonin D can block hydrogen peroxide (H_2_O_2_) induced injuries or apoptosis and is involved in ROS scavenging in human umbilical vein endothelial cells (HUVECs) [12, 13]. Formononetin and extracts of* Schisandra* can evoke vascular relaxation through endothelium-dependent NO pathway [14]. Quercetin from* Astragalus* can improve vascular responsiveness in blood vessels of diabetic rats [15]. Glycyrrhizic Acid from licorice can protect AGE induced endothelial dysfunction in HUVECs [16]. However, the protective effects of HQD on homocysteine induced endothelial dysfunction and the potential mechanisms have not yet been exactly elucidated. Accordingly, we investigate the effects of HQD on endothelium-dependent aortic vasorelaxation and endothelial ROS generation induced by Hcy, thus determining whether the antioxidant mechanism is involved in the beneficial effects of HQD against Hcy-induced endothelial dysfunction.

## 2. Materials and Methods

### 2.1. Reagents and Materials

The herbs were provided by Shanghai Huayu Chinese Herbs Co. Ltd. (Shanghai, China). Dihydroethidium (DHE) was purchased from Molecular Probes Inc. (Eugene, USA). Antibodies of p47phox, p67phox, p40phox, p22phox, and gp91phox were from Santa Cruz Biotechnology (Santa Cruz, CA, USA); Ras-1 and *β*-actin were obtained from Cell Signaling Technology (Danvers, MA, USA); eNOS and p-eNOS were provided by Abcam (Cambridge, MA, USA). Goat anti-rabbit secondary antibodies were bought from Wuhan Boster Biotech Co. Ltd. (Wuhan, China). ECL developer was from Millipore (Billerica, MA, USA). BCA protein quantity kits and PVDF membranes were provided by Pierce (Rockford, IL, USA). Phenylephrine (PE), acetylcholine (ACh), Hcy, sodium nitroprusside (SNP), tempol, apocynin, and N-nitro-L-arginine methyl ester (L-NAME) were purchased from Sigma Chemical Co. Ltd. (St. Louis, MO, USA). All reagents available were of high purity.

### 2.2. Preparation of HQD

HQD was composed of crude herbs including* Astragalus*, Poria,* Trichosanthes* root,* Ophiopogon*,* Schisandra*, licorice, and* Rehmannia* with the ratio of 2 : 2 : 2 : 2 : 1 : 1 : 3 (dry weight). The medicinal herb mixture was extracted three times by four times volume of water, and then the concentration of thick decoction was adjusted to 70% with ethanol overnight. Then the collected supernatant was dried in a drying oven at 105°C for 2 days to obtain dry decoction.

### 2.3. Identification of the Main Compounds in HQD

To identify the active constituents in HQD, the extract was weighed and dissolved in acetonitrile at a concentration of 100 mg/mL. The solution was analyzed by the LC-MS/MS method. LC-MS/MS was conducted on a Shimadzu 30AD HPLC system connected with an Applied Biosystem Sciex Qtrap 5500 mass spectrometer equipped with an ESI ionization source. According to previous reports on HQD, we determined 9 major components (Schisandrin, Liquiritin, Acteoside, Glycyrrhizic Acid, Astilbin, Astragaloside IV, Ruscogenin, Catalpol, and 3,29-dibenzoyl rarounitriol) in HQD. A Thermo Betasil C18 column (50 × 2.1 mm, 5.0 *μ*m) was used for separating Schisandrin, Liquiritin, Acteoside, Glycyrrhizic Acid, Astilbin, Astragaloside IV, and Ruscogenin. A Phenomenex Gemini C18 column (50 × 4.6 mm, 5.0 *μ*m) was used for Catalpol. A Thermo Hypersil C18 column (50 × 2.1 mm, 5 *μ*m) was used for 3,29-dibenzoyl rarounitriol. The mobile phase for separation of Schisandrin, Liquiritin, Acteoside, Glycyrrhizic Acid, Astilbin, Astragaloside IV, Ruscogenin, and Catalpol consisted of 0.1% formic acid in water (A) and 0.1% formic acid in acetonitrile (B) with gradient elution at the flow rate of 0.6 mL/min. The mobile phase for separation of 3,29-dibenzoyl rarounitriol was 10 mM ammonium acetate and 0.1% formic acid in water (A) and methanol (B) with gradient elution at the flow rate of 0.6 mL/min. The gradient elution program was set as follows: 0–0.1 min 5% B, 0.1–1.3 min 5–95% B, 1.3–1.7 min 95% B, 1.7–1.8 min 95–5% B, and 1.8–3.0 min 5% B for Schisandrin, Liquiritin, Acteoside, Glycyrrhizic Acid, Astilbin, and Astragaloside IV; 0–0.1 min 2% B, 0.1–1.3 min 2–98% B, 1.3–1.7 min 98% B, 1.7–1.8 min 98–2% B, and 1.8–3.0 min 2% B for Ruscogenin; 0–0.1 min 30% B, 0.1–1.3 min 30–95% B, 1.3–1.7 min 95% B, 1.7–1.8 min 95–30% B, and 1.8–3.0 min 30% B for Catalpol; 0–0.1 min 5% B, 0.1–1.3 min 5–98% B, 1.3–1.7 min 98% B, 1.7–1.8 min 98–5% B, and 1.8–3.0 min 5% B for 3,29-dibenzoyl rarounitriol.

For MS/MS analysis, optimized parameters were as follows: curtain gas, gas 1, and gas 2 were 15, 40, and 30 psi, respectively; source temperature was 550°C; and spray voltage was 5500 V. The detections were performed in multiple reaction monitoring (MRM) mode, and ion transitions were set at* m/z* 433.4→*m/z* 384.3,* m/z* 417.4→*m/z* 255.1,* m/z* 449.3→*m/z* 150.7,* m/z* 684.5→*m/z* 527.5,* m/z* 623.2→*m/z* 161,* m/z* 821.5→*m/z* 351.1,* m/z* 829.4→*m/z* 783.5,* m/z* 431.5→*m/z* 287.3, and* m/z* 361.4→*m/z* 169 for Schisandrin, Liquiritin, Astilbin, 3,29-dibenzoyl rarounitriol, Acteoside, Glycyrrhizic Acid, Astragaloside IV, Ruscogenin, and Catalpol, respectively.

### 2.4. *Ex Vivo* Isometric Tension Measurement

Male Wistar rats (180–250 g) were from Slaccas Co. Ltd. (Shanghai, China). The study protocols were reviewed and approved by the Ethics Committee of Putuo Hospital, Shanghai University of Traditional Chinese Medicine. Rats were anesthetized with sodium pentobarbital. The thoracic aorta was rapidly removed and placed in cold Krebs solution (pH 7.4) of the following composition: 118.0 g NaCl, 4.7 g KCl, 1.2 g MgCl_2_·6H_2_O, 1.2 g KH_2_PO_4_, 25.0 g NaHCO_3_, 2.5 g CaCl_2_, and 11 g glucose. The vessels were dissected to be devoid of adipose and connective tissues and cut into rings at the length of 3 mm. Endothelium was removed by gently rubbing the intimal space with a toothpick. The aortic rings were suspended by two L-shaped stainless steel wires inserted into the lumen, in a 10 mL organ chambers filled with Krebs solution, maintained at 37°C and oxygenated continuously with 95% O_2_ and 5% CO_2_. The isometric tension was recorded with a force transducer and Power Lab recording system (ADInstruments Pty Ltd). Rings were equilibrated for 60 min at 3 g. The bath solution was changed every 20 min before exposing arteries to KCl (60 mM). After a further equilibration period of 30 min, the integrity of the endothelium was assessed by determining the ability of ACh (10^−6^ M) to induce more than 80% relaxation of rings precontracted with PE (3 × 10^−7^ M). The endothelium was considered to be removed when there was less than 10% relaxation response to ACh.

To assess the effects of HQD on isolated aortic rings with intact endothelium precontracted with PE (3 × 10^−7 ^M) and determine the dosage used in the subsequent experiments, HQD at a dosage ranging from 0.1 to 1000 *μ*g/mL was cumulatively added into the organ bath with 5 min interval. The rings with intact endothelium were tested in parallel without drug as the control.

To assess the effect of Hcy (1 mM, 1 h) on endothelium-dependent or endothelium-independent relaxation, ACh (10^−10^–10^−4 ^M) or SNP (10^−11^–10^−5 ^M) was added after PE (3 × 10^−7 ^M) induced contraction. To investigate the role of ROS in the observed inhibitory effect of Hcy, rings were coincubated with tempol (100 *μ*M), apocynin (100 *μ*M), and Hcy (1 mM). Furthermore, to determine whether HQD has similar effects with tempol and apocynin, rings were treated with HQD (10, 30, and 100 *μ*g/mL) instead of tempol and apocynin. In addition, to determine whether increasing NO production is involved in the protective effect of HQD, tempol, and apocynin on impaired endothelium-dependent relaxation induced by Hcy, aortic rings were pretreated with L-NAME (100 *μ*M, 30 min) before PE-induced contraction.

### 2.5. Isolation of Endothelial Cells

Human umbilical vein endothelial cells (HUVECs) were digested from fresh newborn umbilical cords as previously described [17]. Briefly, perfuse veins of umbilical cords with PBS (pH 7.4, 0.2 M) to discard blood cells. Then fill them with 0.1% collagenase for 15 min at 37°C. Thereafter, collect the digestion and centrifuge at 1000 rpm for 5 min. HUVECs were cultured in basal Dulbecco's modified Eagle's medium (DMEM) supplemented with 20% fetal bovine serum (FBS, Gibco, USA), endothelial cell growth supplement (ECGS), and antibiotics. HUVECs from passages 3-4 were used in the subsequent experiments.

### 2.6. Measurement of ROS Generation

ROS generation was evaluated using fluorescence probe DHE according to the protocol. After successive treatment with HQD or antioxidants for 30 min and Hcy for another 30 min, the supernatant was used for analyzing oxidative stress markers including superoxide dismutase (SOD), malondialdehyde (MDA), lipid-peroxides (LPO), and hydrogen peroxide (H_2_O_2_) through assay kits (Nanjing Jiancheng Bioengineering Institute, Nanjing, China). HUVECs were continually incubated with the probe DHE for 30 min at 37°C. Then cells were washed with PBS to remove free DHE molecules, fixed with paraformaldehyde (10 *μ*g/mL). Fluorescence was monitored via a fluorescence microscope (Nikon TE2000, Japan).

### 2.7. Measurement of NO Production and Phosphorylation of eNOS

HUVECs were seeded in 24-well plate until 90% confluence. The cells were incubated with HQD (10, 30, and 100 *μ*g/mL), tempol (100 *μ*M), and apocynin (100 *μ*M) for 30 min, after which Hcy (1 mM) was incubated for another 60 min. Then the supernatants were collected for the determination of extracellular NO level by assay kits (Nanjing Jiancheng Bioengineering Institute, Nanjing, China). The cells were washed with ice-cold PBS and collected for the extraction of total protein with RIPA lysis buffer. The concentration of extracted protein was determined using BCA kits. Then SDS-PAGE was performed with 20 *μ*g of protein sample to determine the expression of eNOS and p-eNOS.

### 2.8. Measurement of NADPH Oxidase Production

HUVECs were seeded in 24-well plate until 90% confluence. After treatment with HQD (10, 30, and 100 *μ*g/mL), tempol (100 *μ*M), and apocynin (100 *μ*M) for 30 min, HUVECs were incubated with Hcy (1 mM) for another 60 min. Then the supernatants were collected for the determination of extracellular NADPH oxidase levels by assay kits (Wuhan ColorfulGene Biological Technology Co., Ltd., Wuhan, China). The extracted proteins from HUVECs underwent western blot analysis to determine the expression of NADPH oxidase subunits including p47phox, p67phox, p40phox, p22phox, gp91phox, and Ras-1.

### 2.9. Statistical Analysis

Responses to ACh and SNP were represented as % of the maximal contraction to PE. The concentration of ACh that induced 50% of the maximal relaxation was regarded as the EC50 which was determined by regression analysis of the log dose-response curves. EC50 was expressed as pD2 (−log EC50). All data were expressed as mean ± SEM. Statistical analysis was performed via Student's unpaired *t*-test between two groups or One-Way Analysis of Variance (ANOVA) with Tukey's* post hoc* test among more than two groups (GraphPad Prism 5.0 software, GraphPad Prism software Inc., San Diego, California, USA). A value of *P* < 0.05 was judged to be significant.

## 3. Results

### 3.1. Identification of the Compounds in HQD Extract

**Figure 1:**
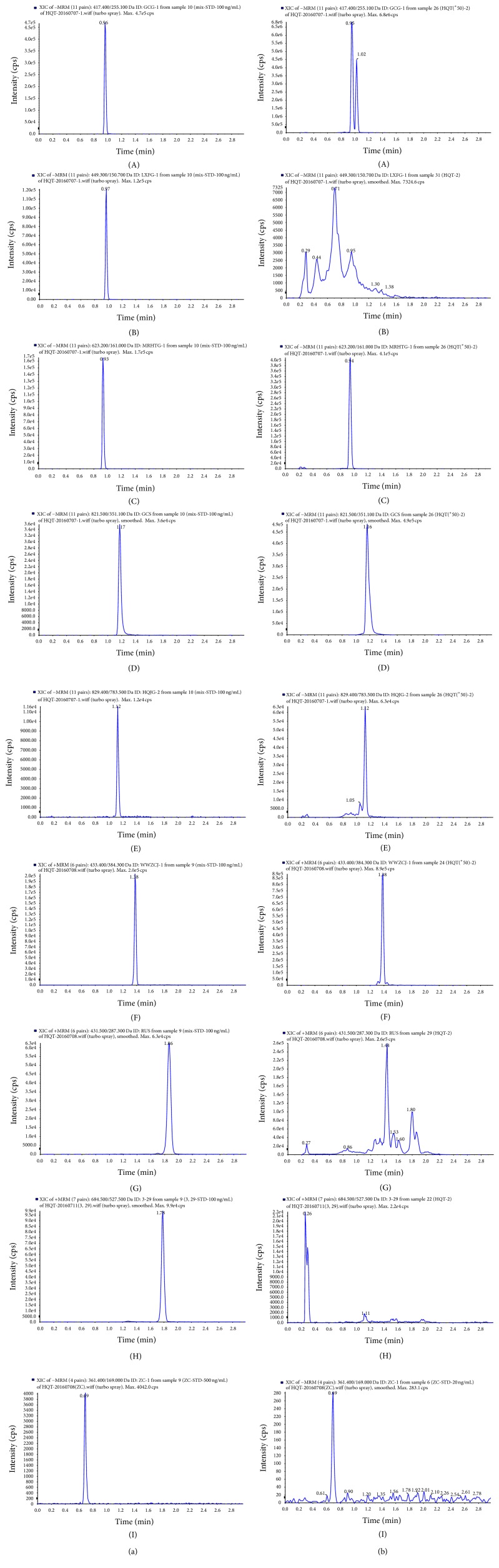
The extracted ion chromatograms of the detected components in HQD. Reference standard (a) and sample (b). (A) Liquiritin, (B) Astilbin, (C) Acteoside, (D) Glycyrrhizic Acid, (E) Astragaloside IV, (F) Schisandrin, (G) Ruscogenin, (H) 3,29-dibenzoyl rarounitriol, and (I) Catalpol.

Nine main constituents were analyzed using LC-MS/MS method. Except for 3,29-dibenzoyl rarounitriol, all other eight components could be detected in HQD and further quantified. The ion chromatograms of these compounds were presented in Figure 1. The content of Liquiritin, Astilbin, Acteoside, Glycyrrhizic Acid, Astragaloside IV, Schisandrin, Ruscogenin, and Catalpol in HQD extract was 1047.69, 0.04, 150.46, 879, 337.82, 264.69, 0.78, and 0.32 *μ*g/g, respectively.

### 3.2. Effect of HQD on Vasomotor Impairment Induced by Hcy

**Figure 2:**
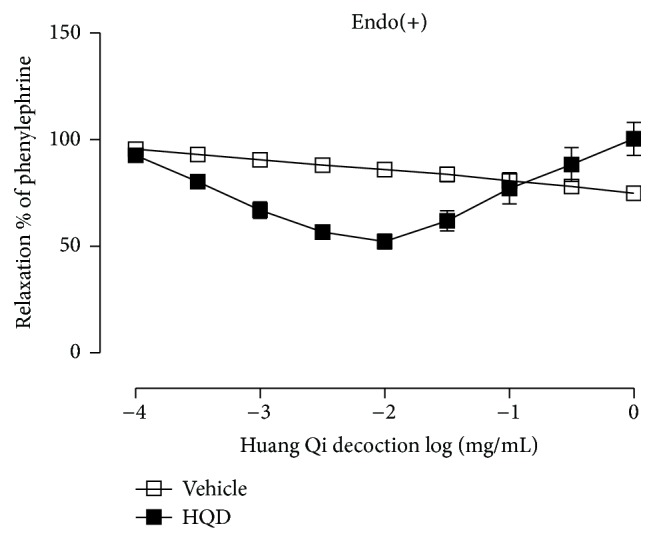
Effects of Huang Qi decoction on phenylephrine-induced contractions in aortic rings with endothelium.

**Figure 3:**
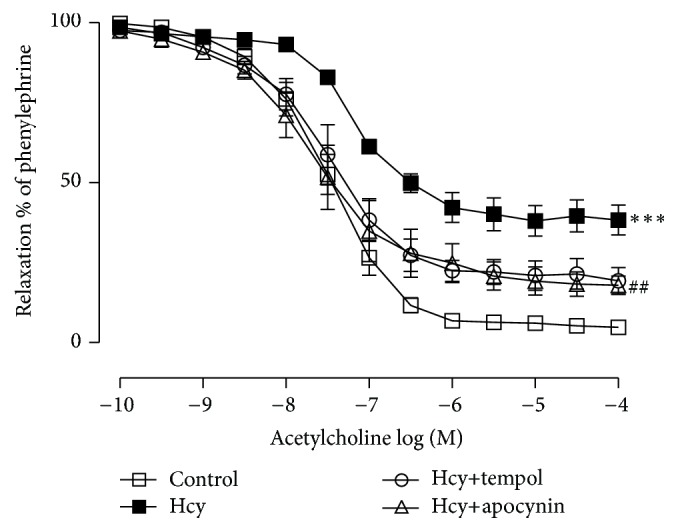
Inhibitory effect of homocysteine (Hcy) on the endothelium-dependent relaxation and improvement caused by tempol and apocynin in aortic rings. Values are means ± SEM (*n* = 6). ^*∗∗∗*^ *P* < 0.001 compared with the control group and ^##^ *P* < 0.01 compared with the Hcy group.

As shown in Figure 2, in PE-precontracted endothelium-intact rings, low doses of HQD showed relaxation while high dose larger than 100 *μ*g/mL might induce contraction. In Figure 3, in any group, no difference in relaxation responses to ACh (10^−6 ^M) in aortic rings was observed at the initial experiment. Compared with the control group (*E*_max_: 95.19 ± 0.97% and pD2: 7.46 ± 0.10), pretreatment with Hcy for 60 min reduced ACh-induced relaxation according to the data that *E*_max_ fell to 64.15 ± 5.11%, with a reduction of pD2 (7.12 ± 0.08). Endothelium-independent relaxation to SNP was unaffected by Hcy (data not shown).

**Figure 4:**
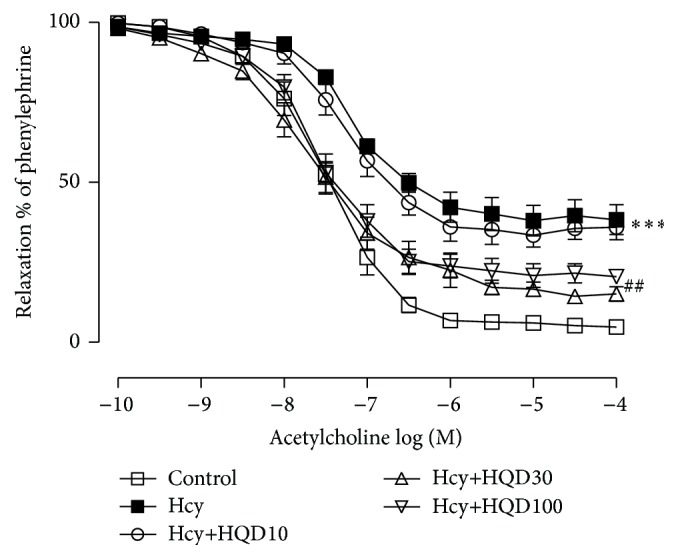
Improvement caused by Huang Qi decoction on homocysteine- (Hcy-) induced endothelium-dependent vasorelaxation impairment in aortic rings. Values are means ± SEM (*n* = 6). ^*∗∗∗*^ *P* < 0.001 compared with the control group and ^##^ *P* < 0.01 compared with the Hcy group.

Cotreatment of HQD (30 and 100 *μ*g/mL) with Hcy was able to improve the inhibition of endothelium-dependent relaxation induced by Hcy. *E*_max_ and pD2 were shown as 85.89 ± 2.20% or 82.81 ± 2.51% versus 64.15 ± 5.11% (*P* < 0.01) and 7.63 ± 0.16 or 7.59 ± 0.05 versus 7.12 ± 0.08 (*P* < 0.05 or *P* < 0.01) (Figure 4).

To confirm the role of ROS in the observed inhibitory effect on endothelium-dependent relaxation by Hcy, tempol and apocynin were used. In Figure 2, *E*_max_ and pD2 were shown as 81.87 ± 2.05% or 84.80 ± 1.62% versus 64.15 ± 5.11% (*P* < 0.01) and 7.49 ± 0.17 or 7.56 ± 0.20 versus 7.12 ± 0.08.

**Figure 5:**
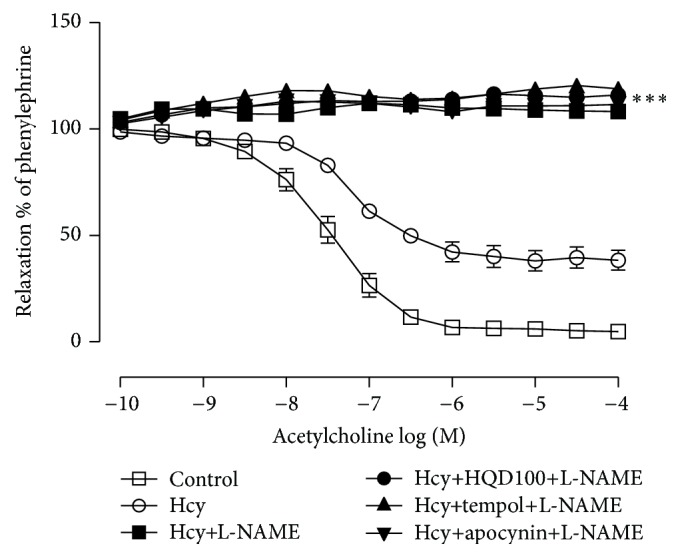
Effect of N-nitro-L-arginine methyl ester (L-NAME) on the protection of Huang Qi decoction (HQD), tempol, and apocynin against the impairment of endothelium-dependent relaxation induced by homocysteine (Hcy). Values are means ± SEM (*n* = 6). ^*∗∗∗*^ *P* < 0.001 compared with the Hcy group.

As presented in Figure 5, incubation of aortic rings with L-NAME (100 *μ*M, 30 min) abolished the improvement of HQD (100 *μ*g/mL), tempol (100 *μ*M), and apocynin (100 *μ*M) on endothelium-dependent relaxation.

### 3.3. Effect of HQD on Intracellular ROS Stimulated by Hcy

**Figure 6:**
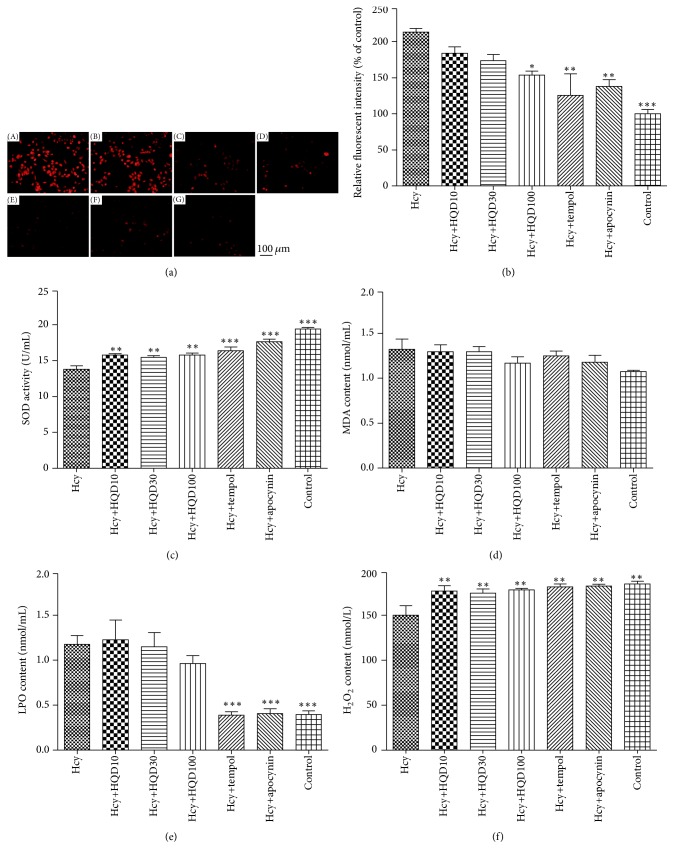
(a) ROS generation was examined by DHE staining. Bar represented 100 *μ*m. (A) Hcy group; (B) Hcy+HQT10 group; (C) Hcy+HQT30 group; (D) Hcy+HQT100 group; (E) Hcy+tempol group; (F) Hcy+apocynin group; and (G) control group. (b) Relative fluorescence intensity of intracellular ROS. (c) SOD activity. (d) MDA content. (e) LPO content. (f) H_2_O_2_ content. Data are shown as means ± SEM, *n* = 3; HQD: Huang Qi decoction; control: Hcy and HQD untreated group; Hcy: HUVECs treated with Hcy; HQD10: HUVECs group treated with Hcy and HQD at a concentration of 10 *μ*g/mL; HQD30: HUVECs group treated with Hcy and HQD at a concentration of 30 *μ*g/mL; HQD100: HUVECs group treated with Hcy and HQD at a concentration of 100 *μ*g/mL; tempol: HUVECs group treated with Hcy and tempol; and apocynin: HUVECs group treated with Hcy and apocynin. ^*∗*^ *P* < 0.05, ^*∗∗*^ *P* < 0.01, and ^*∗∗∗*^ *P* < 0.001 versus the Hcy group.

Effect of HQD on ROS provocation was determined by DHE fluorescence staining method in HUVECs. As presented in Figures 6(a) and 6(b), Hcy substantially increased DHE fluorescent density, whose effect was eliminated by the treatment of tempol or apocynin. Hcy-provoked ROS production was significantly inhibited by 28% with the pretreatment of HQD of 100 *μ*g/mL compared with Hcy group. HQD at the dose of 10 or 30 *μ*g/mL did not significantly influence DHE fluorescent intensity. There was not any significant difference in the level of MDA between all the seven groups. The SOD activity and content of H_2_O_2_ in Hcy group were significantly decreased compared with the control group and such decrease was reversed by treatment with HQD, tempol, and apocynin. LPO content was markedly increased in the Hcy group, which was decreased by tempol and apocynin but not by HQD (Figures 6(c)–6(f)).

### 3.4. Effect of HQD on NO Production

**Figure 7:**
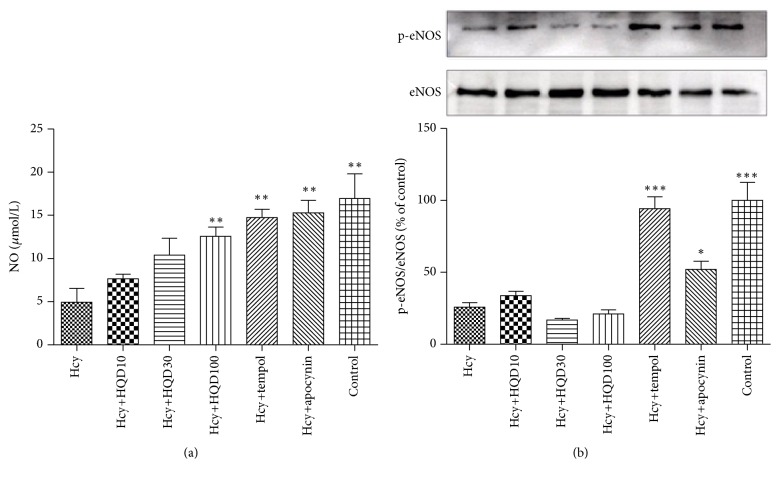
(a) NO production. (b) Phosphorylation of eNOS. Data are shown as means ± SEM, *n* = 3; HQD: Huang Qi decoction; control: Hcy and HQD untreated group; Hcy: HUVECs treated with Hcy; HQD10: HUVECs group treated with Hcy and HQD at a concentration of 10 *μ*g/mL; HQD30: HUVECs group treated with Hcy and HQD at a concentration of 30 *μ*g/mL; HQD100: HUVECs group treated with Hcy and HQD at a concentration of 100 *μ*g/mL; tempol: HUVECs group treated with Hcy and tempol; and apocynin: HUVECs group treated with Hcy and apocynin. ^*∗*^ *P* < 0.05, ^*∗∗*^ *P* < 0.01, and ^*∗∗∗*^ *P* < 0.001 versus the Hcy group.

As expected in Figure 7(a), the addition of Hcy significantly reduced NO production by 71% in comparison with the control group, which was considerably reversed by the pretreatment of tempol or apocynin. The application of HQD at the dose of 100 *μ*g/mL significantly attenuated the decrease of NO level induced by Hcy. However, HQD at the dose of 10 or 30 *μ*g/mL exerted nonsignificant effects on Hcy-induced reduction of NO.  Figure 7(b) presented that HQD did not influence phosphorylation of eNOS.

### 3.5. Effect of HQD on NADPH Oxidase Production

**Figure 8:**
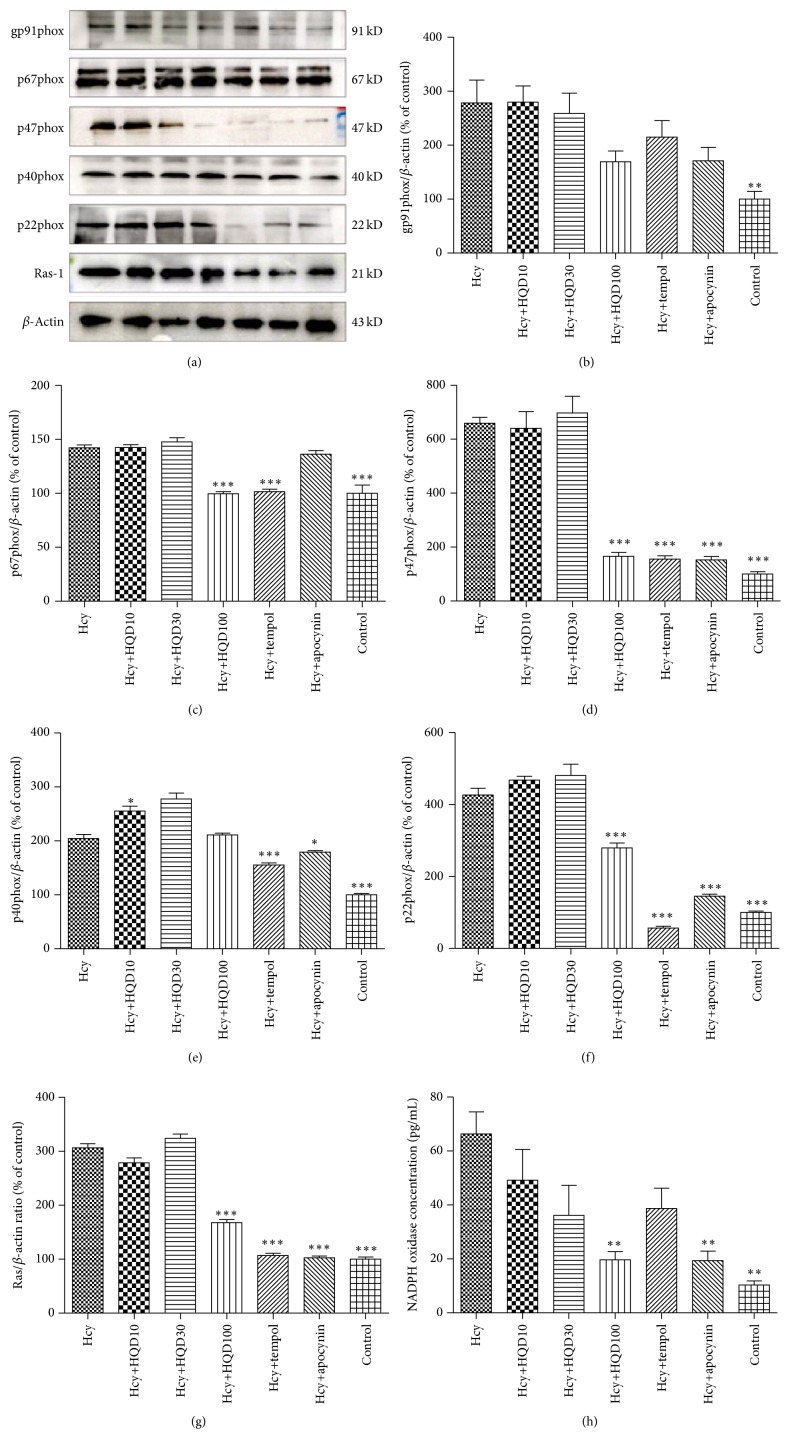
Effects of HQD on the intracellular and extracellular concentration of NADPH oxidase. (a) Western blot for NADPH oxidase subunits including gp91phox, p67phox, p47phox, p40phox, p22phox, and Ras-1. (b)–(g) Statistical analysis of (a). (h) Extracellular concentration of NADPH oxidase. HQD: Huang Qi decoction; normal: Hcy and HQD untreated group; Hcy: HUVECs treated with Hcy; HQD10: HUVECs group treated with Hcy and HQD at a concentration of 10 *μ*g/mL; HQD30: HUVECs group treated with Hcy and HQD at a concentration of 30 *μ*g/mL; HQD100: HUVECs group treated with Hcy and HQD at a concentration of 100 *μ*g/mL; tempol: HUVECs group treated with Hcy and tempol; and apocynin: HUVECs group treated with Hcy and apocynin. ^*∗*^ *P* < 0.05, ^*∗∗*^ *P* < 0.01, and ^*∗∗∗*^ *P* < 0.001 versus the Hcy group.

Western blot monitored the expression levels of NADPH oxidase subunits including Ras-1, p47phox, p67phox, p40phox, p22phox, and gp91phox. As shown in Figure 8, HUVECs treated with Hcy alone presented the highest expression level of Ras-1, p47phox, p67phox, p40phox, p22phox, and gp91phox, respectively, which could be decreased by tempol or apocynin treatment. Compared with Hcy group, HQD inhibited the expression of NADPH oxidase subunits. In terms of inhibitory efficacy of HQD at different dosage, HQD at the dose of 100 *μ*g/mL exhibited the best performance.

## 4. Discussion

HQD was a classic traditional Chinese medical formula, whose components had been demonstrated to exert effective effects on diabetic complications through antioxidative mechanism [6, 18]. In our study, we demonstrated that HQD at an appropriate dosage had potential to treat diabetic vascular complications by improving impaired vasodilation and endothelial function through attenuating oxidative damage. It decreased the expression of NADPH oxidase, inhibited the production of ROS, and increased production of NO, thereby promoting vascular endothelial function of aortic rings.

Oxidative damage was one of the pivotal contributors to diabetic vascular complications and endothelial dysfunction [19]. Previous investigations had addressed the fact that elevated Hcy concentrations would cause vascular endothelium damage via overgenerating oxygen-derived free radicals, in a sense, namely, ROS, which were formed via oxidation of the sulfhydryl base group in Hcy [20, 21]. In the present study, we used Hcy at a concentration of 1 mM, which represented approximately 10-fold dose of clinical plasma levels of severe hyperhomocysteinemia (>100 *μ*M). Vascular dilation of preconstricted vessels in response to ACh was endothelium dependent. Our results showed that exposure of intact vessels to Hcy remarkably decreased ACh-induced vasorelaxation, implicating that Hcy would cause endothelium damage. To explore the role of ROS, we used tempol, a putative superoxide dismutase (SOD) mimetic which could scavenge superoxide anions. As expected, tempol significantly reversed the increased superoxide anions generation and LPO levels in Hcy group. Additionally, cells were equipped with antioxidant defense including SOD, which could convert superoxide anions to H_2_O_2_. Tempol increased activity of antioxidants SOD which was indicated by elevated levels of H_2_O_2_ compared with Hcy group. Considering these indicative markers of oxidative stress, tempol improved Hcy-induced endothelium-dependent vasodilatory impairment, indicating that ROS might be involved in Hcy-induced endothelium damage. A major source and modulatory enzyme of ROS in the vasculature was NADPH oxidase, a multisubunit enzyme which was composed of membrane-associated cytochrome b558 including p22phox and gp91phox subunits and cytoplasmic subunits including p47phox, p67phox, p40phox, and the small G protein Rac [22]. To exactly explore whether NADPH oxidase derived ROS played a role, apocynin, an NADPH oxidase inhibitor, was applied [23]. Compared with Hcy, apocynin effectively decreased NADPH oxidase expression and ROS production and dilated arterial vessels* ex vivo*, putatively verifying that NADPH oxidase-dependent ROS generation might exert effects on Hcy-induced impaired vasodilation. Thus antioxidative strategy might improve Hcy-induced endothelial dysfunction.

HQD mainly contained* Astragalus* (Huang Qi), Poria (Fu Ling),* Trichosanthes* (Gua Lou),* Ophiopogon* (Mai Dong),* Schisandra* (Wu Wei Zi), licorice (Gan Cao), and* Rehmannia* (Di Huang). These components had been reported to exhibit antioxidant pharmacological properties in various tissues and cells. Accordingly, we suspected that HQD might have potential to ameliorate vascular endothelial dysfunction. In our study, HQD at a dosage larger than 100 *μ*g/mL would induce contraction in PE-precontracted endothelium-intact rings, while HQD at the concentrations ranging from 10 to 100 *μ*g/mL suppressed the contraction induced by PE in aortic rings with endothelium. Such result was observed in another research in which the treatment with high dose of Huang Qi increased vascular tone [24]. Therefore concentrations of HQD ranging from 10 to 100 *μ*g/mL were applied in our subsequent experiments. As expected, HQD at a proper dosage of 30 or 100 *μ*g/mL significantly reversed the inhibitory effects of Hcy on ACh-induced vasorelaxation. Moreover, HQD at an appropriate dosage significantly restored the increased superoxide anions and decreased SOD induced by Hcy. Meanwhile, HQD at a dosage of 100 *μ*g/mL decreased the expression of NADPH oxidase, which was one of the sources of ROS. This coordinately demonstrated that there existed an association between the protective effect against Hcy-induced endothelial dysfunction and the inhibitory activity against Hcy-induced increased expression of NADPH oxidase and NADPH oxidase derived ROS. Similar antioxidant activity had been observed in previous reports. In these studies,* Astragalus*, the component of HQD, was documented to block oxidative stress-induced apoptosis in kidney epithelium [25]. Wojcikowski et al. also demonstrated that 55 kinds of medicinal herbs, including the constituents of HQD such as* Astragalus* and licorice, had an antioxidant capacity [26]. Therefore, the protective effects of HQD on Hcy-triggered endothelial dysfunction might be attributed to the antioxidative activity of its components. However, the components responsible for the protective performance need to be further explored.

NO was a major regulatory factor of vascular relaxation and endothelial function [27]. In our investigation, an inhibitor of NO synthase, L-NAME, suppressed the improved effects of HQD of 100 *μ*g/mL on Hcy-induced impaired vasorelaxation. Such observation suggested that protective effects of HQD on Hcy-induced vascular endothelial dysfunction were likely to be linked with improved bioavailability of endothelial NO. Actually, Hcy treatment exactly declined NO production as well as the phosphorylation of eNOS which regulated NO generation. HQD at a dose of 100 *μ*g/mL increased production of NO in comparison with Hcy treatment and HQD at the dose 10 or 30 *μ*g/mL showed similar effects to Hcy. Hcy-triggered overgeneration of ROS in the vascular endothelium, which were formed via oxidation of the sulfhydryl base group in Hcy, had been considered to impair bioavailability of endothelium derived NO, resulting in impaired vasodilation [21]. On one hand, ROS could react with NO to consume NO and form a kind of deleterious oxidative molecule, which would further cause oxidative injury in endothelial cells. On the other hand, endothelial NO synthase (eNOS) might be oxidized or inhibited from activation, thus negatively influencing the derivation of NO. Both of abovementioned phenomena would contribute to the decreased NO bioavailability and impaired endothelial NO mediated vasodilation. With the antioxidative activity, HQD at an appropriate dosage could alleviate impaired NO bioavailability induced by Hcy, thus improving impaired vasodilation.

An impairment of NO mediated endothelium-dependent relaxation and an enhanced generation of ROS and NADPH oxidase were observed following Hcy exposure. Therefore, regulating the activity of eNOS and production of NO via controlling the formation of ROS might be a key strategy for attenuation of endothelial dysfunction induced by Hcy. In the current study, HQD could induce production of NO and decrease ROS through attenuation of ROS-producing enzymes, respectively. Therefore, HQD could alleviate Hcy-induced endothelial dysfunction. However, we have not addressed the ROS deriving from all of vascular oxidase, but focused on ROS production involving NADPH oxidase.

## 5. Conclusion

The current study indicated that HQD exerted a favorable effect on Hcy-induced impaired vasomotor response of aortic rings and oxidative stress in HUVECs via partial antioxidant mechanism. Collectively, HQD at a proper dosage provides an alternative for amelioration of oxidative stress-associated endothelial dysfunction, thereby potentially preventing diabetic related vascular disorders.
